# Full Blood Count and its Differentials in Acute Aortic Dissection: An Update and Future Perspectives

**DOI:** 10.1055/a-2717-6200

**Published:** 2025-10-29

**Authors:** Panagiota Georgiadou, John Elefteriades

**Affiliations:** 1 Division of Interventional Cardiology, Onassis Cardiac Surgery Center, Athens, Greece; 2 Aortic Institute at Yale New Haven Hospital, Yale University School of Medicine, New Haven, Connecticut, United States

**Keywords:** acute aortic dissection, biomarkers, full blood count, diagnosis, aneurysm

## Abstract

Acute aortic dissection (AAD) is a potentially lethal condition with a high rate of misdiagnosis during the initial evaluation. In addition to established clinical variables, previous studies have consistently demonstrated a relationship between full blood count (FBC) and its various differentials with acute aortic syndromes, even identifying patients with worse outcome. Although FBC is a simple, inexpensive and routinely performed test, it is easily overlooked by clinicians. However, nearly all components of FBC, including white blood count, red blood cells, and platelets, may contribute to the underlying pathogenesis of AAD and therefore, we should fully explore and pinpoint precisely their potential diagnostic or prognostic performances. Herein, we summarize the results of such studies and discuss controversies regarding utility in future clinical practice.

## Introduction

Acute aortic dissection (AAD) is a lethal cardiovascular emergency with a mortality rate of 1 to 2% per hour early after symptom onset. 1 2 Ninety-five percent of AAD patients are asymptomatic before this event occurs; furthermore, even 20% of symptomatic patients may have inconclusive signs and symptoms. 1 2 Unfortunately, in 50% of the emergency department cases, the AAD may be misdiagnosed initially, even in the present era, adversely affecting clinical outcomes. Furthermore, the increasing use of imaging in the emergency department (ED) has not substantially improved misdiagnosis rates, indicating that pretest patient selection remains crucial.

In the highly heterogenous group of clinical cases with potential AAD, the majority are classified as low probability acute aortic syndromes (AAS). The standardized interpretation of biomarkers along with bedside echocardiography and/or plain chest X-ray represent an acceptable method of fine-tuning the pretest probability assessment. 3 Although a number of single new biochemical markers have been assessed for initial diagnostic screening, the majority of such tests are nonspecific in detecting AAD—and often—not readily available and expensive. 4

On the other hand, routine full blood count (FBC) and its differentials may reflect multiple aspects of the true complexity of aortic dissection. However, the FBC reflects not only the variety of pathophysiologic pathways activated in AAD (inflammation, coagulation, and immune response), but it can also reveal the systemic responses of concomitant malperfused organs. 5 Nevertheless, these traditional hematological parameters, despite the ease of their acquisition, continue to be regularly underappreciated by clinicians in their everyday practice. 5 Careful interpretation of the abnormalities in FBC and blood count-derived biomarkers, individually or in combination, along with clinical data and first-line imaging tools, may help to establish pretest probability for AAS.

This review summarizes the available literature on the diagnostic performance of FBC and its differentials in outpatients or patients admitted to an ED with suspected AAD. We hope that this overview can be useful to identify clinical knowledge gaps and also to guide future research in this field.

### White Blood Cells

#### White Blood Cell Count

White blood cell count (WBC) is a sensitive but nonspecific inflammatory marker, almost universally employed in seriously ill patients. Multiple studies have corroborated that WBC is an independent predictor of cardiovascular events and in-hospital mortality in AAD patients. 6 7 8 9 10 11 Elevated admission WBC levels have been associated with AAD from its development stage up to outcome prediction. WBC levels reflect both the acute phase reaction as well as the underlying inflammatory process, quantifying in a way, the extent of aortic injury.

Most studies have shown significantly increased WBC levels in AAD patients compared with other chest pain (CP) groups like pulmonary embolism, acute coronary syndromes, pericarditis, presenting within 48h from the onset of symptoms. Conversely, C-reactive protein (CRP) values were not as useful ( Table 1 ). 6 7 8 9 10 11 Sbarouni et al. also found higher WBC in patients with AAD compared with chronic aneurysms and to controls (13044 ± 4885 vs. 7833 ± 1628 K/μL vs. 6983 ± 2252 K/μL, *p* = 0.0005). On the other hand, CRP was significantly higher in both AAD and chronic aneurysms compared with controls; but CRP did not distinguish between acute and chronic aortic disease patients ( *p* = 0.1; Table 1 ). 10 However, when the receiver operating characteristic (ROC) curve of WBC was used for diagnosis of AAD, its diagnostic value was markedly lower than that of D-Dimers ( *p* < 0.0001).

**Table 1:** Summary of studies investigating white blood cell in AAD

	Sample size	Study design	Mean age	Gender M/F	Duration of symptoms	White blood cell			*p* -Value
						AAD	Aneurysms	Controls	
Eggebrecht et al 2004	16	Monocentric, retrospective, observational	65.2 ± 15.2 (36–83)	11/5	16 ± 15.7 h (2–48)	14.6 ± 5.0 (7.0–25.6)	8.3 ± 2.3 (4.5–15.1) *N* = 32 chronic stable dissection	7.7 ± 2.1 (4.4–12.5) *N* = 16 non-cardiac chest pain pts	<0.001
Hazui et al 2005	29	Monocentric, retrospective, case–control	65 ± 11	16/13	4 h	12.7 ± 4.9		11,673 ± 3,672.5, *N* = 49 pts with AMI	0.363
Ohlmann et al 2006	92	Single center, retrospective, case–control	63.8 ± 12.6	62/32	1.2 ± 2.5 d	10.7 (IQR: 9.32; 13.1)		9.7 (6.9; 12.5) *N* =87 patients with other diagnosis	0.01
Morello et al 2015	271	Monocentric, retrospective, observational	67.5 ± 13.7	195/76		11.0 (IQR: 8.8–13.3)		8.3 (IQR 6.6–10.8) patients with other diagnosis	0.001
Sbarouni et al 2007	18	Monocentric, retrospective, observational	53 ± 17 (18–78)	17/1	17 ± 15 (4–60) h	13 ± 4.8 (6.3–23.3)	7.8 ± 1.6 (4.3–11) *N* = 21	6.9 ± 2.2 (3.8–10.6) *N* = 8 healthy controls	0.0005
Li et al 2013	31	Monocentric, prospective, observational			First week	11.19 ± 4.97		6.40 ± 1.66	0.000

Abbreviations: AAD, Acute aortic dissection; WBC, white blood count.

Morello et al showed 67.3% sensitivity and 59% specificity of WBC > 9 × 10 ^3^ /µL among 891 patients classified with low probability for all AAS according to the risk assessment tool proposed in 2010 American College of Cardiology/American Heart Association guidelines (area under the curve [AUC] = 0.69 [95% confidence interval, CI: 0.63–0.74, *p* < 0.001]). 9 12 They also reported that the presence of at least one alteration among, WBC > 9 × 10 ^3^ /µL platelet (PLT) count < 200 ×10 ^3^ /µL and fibrinogen < 350 mg/dL yielded a sensitivity of 95.5% (89.7–98.5%) and a specificity of 18.3% (15.6–21.2%) for AAD. Further in the same study, WBC > 9 × 10 ^3^ /µL and platelet count <200 × 10 ^3^ /µL were found to predict AAS independently of symptoms, pulse deficit, and hypotension. 9

Among the factors reported to be related to WBC levels are the aortic diameter ( *p* = 0.000) and the extent of dissection, with a median (25th, 75th percentile) WBC of 10.4 (8.1, 13.9) × 10 ^3^ /μL for dissection confined to the ascending aorta compared with 13.3 (9.8, 15.9) × 10 ^3^ /μL for extension down to the iliac artery ( *p* < 0.001). 13 14 Wen et al also showed a significant inverse relationship between WBC levels and the time period from the symptom onset to hospital admission (mean time: 3.4 ± 1.7 days, *r* = –0.200, *p* = 0.002). 14 The association between WBC and type of AAD is not consistently reported.

#### Neutrophils

The association between neutrophils and AAD has been consistently observed in different populations. Neutrophilia and neutrophil-related markers have been associated with various mechanisms potentially relevant for AAD, including the expression of proinflammatory triggers, the induction of endothelial cell injury, and prothrombotic states. Sbarouni et al have detected higher WBC and neutrophil percentage in patients with Type A AAD compared with both chronic aortic aneurysms and normal subjects, indicating enhanced immuno-inﬂammatory activity. 10 15 Del Porto et al showed high percentages of neutrophils in peripheral blood, but not inside the aortic wall, suggesting that these cells may not have a direct role in the pathogenesis of aortic rupture but rather be the result of acute stress associated with aortic wall rupture. 16

Chun et al found higher values of neutrophils during the time interval of 2 to 24 hours after symptom onset and reported a sensitivity of 94.6% (95% CI: 84.2–98.6%) and a specificity of 52.3% (95% CI: 42.1–61.9%) at a cutoff value of 6.2 × 10 ^9^ /L in the AAD risk score ≤ 1 group compared with other CP patients. 17 Moreover, neutrophil counts had similar good ability to identify AD in patients with different types of AAD and different AAD scores. 12 17 Regarding time-course analysis, the same investigators observed a marked increase in neutrophil counts from 0 to 2 hours and peaking at 2 to 24 hours after the symptoms' onset ([6.4 ± 3.4] ×10 ^9^ /L to [10.3 ± 4.3] × 10 ^9^ /L, *p* < 0.001). 17 The sensitivity and specificity of neutrophil counts reported in the 2- to 8-hour interval was 94.8% (95% CI: 84.7–98.6%) and 59.4% (95% CI: 50.0–68.4%) respectively, with similar sensitivity and specificity in the 8- to 24-hour interval group, comparable to accuracies seen with D-dimers. 17 Further, Li et al demonstrated higher levels of WBC and neutrophils during 1-week, 2-weeks and 4-weeks post-AAD onset compared with coronary heart disease patients, with the highest level occurring during the ﬁrst week ( Table 1 ). 11

#### Lymphocytes, Monocytes, Eosinophils

Previous studies have reported low lymphocyte and elevated monocyte counts in peripheral blood of AAD patients, significantly different compared with those of patients with chronic heart disease and normal volunteers; 11 16 additionally, the highest number of monocytes was detected in AAD patients during the first week. 11 Decreased lymphocyte to monocyte ratio—or, expressed differently as increased monocyte to lymphocyte ratio—are indices of inflammation and immune dysregulation, which have been associated with high incidence of in-hospital mortality in AAD patients. 18 19

Decreased blood eosinophil (EOS) levels were consistently reported in AAD patients compared with healthy controls. 11 Several studies have demonstrated low EOS percentage as independent predictor of in-hospital mortality for AAD patients. 20 21 Extensive EOS infiltration was observed in AD thrombus in the false lumen, indicating the extent of preoperative vascular injury. 21 It is noteworthy that EOS deﬁciency has been presented as a compensatory mechanism with a protective and no pathogenic role in abdominal aortic aneurysms. 22

#### Neutrophil to Lymphocyte Ratio

Neutrophil to lymphocyte ratio (NLR) is an established inflammatory marker that seems to aid in risk stratification of cardiovascular diseases, in addition to traditional markers. 23 NLR, as a ratio index, is more stable than the individual leukocytic parameters, which could be affected by hydration status and sample handling. Elevated NLR, indicative of high neutrophil count due to active inflammation and low lymphocyte count due to defective inflammatory responses, achieved great diagnostic power.

Our study showed NLR to be significantly higher in 120 patients with AAD Type A compared with patients with chronic aneurysms and with age- and sex-matched healthy subjects (10.1 [5.9–14.3] vs. 2.2 [1.7–2.9] vs. 2.0 [1.4–3.1], *p* < 0.001 for all comparisons). 15 NLR was predictive of the diagnosis of AAD with a sensitivity of 89% and a specificity of 91% at a cutoff value above 4.6 at presentation. 15 Similarly, Onuk et al showed that NLR was significantly higher in the AAD group compared with aortic dilatation and control groups (median 8.83 [8.13] vs. median 1.95 [1.10] vs. median 1.71 [0.77], respectively, *p* = 0.01). 24 In another study, Lareyre et al showed that the proportion of patients with pain or with ruptured thoracic aortic aneurysm was significantly higher in patients with an NLR > 3.5 compared with those having NLR < 3.5 (42.1 vs. 16.2%; *p* = 0.022 and 26.3 vs. 2.7%; *p* = 0.007, respectively), basically due to the combined neutrophilia and lymphopenia, compared with those with neutrophilia and lymphopenia alone. 25 In a larger sample size, Zhang et al found significantly elevated NLR levels in AAD patients compared with chronic dissected aneurysms and other acute CP diseases (11.79 [6.39–16.54], 4.83 [2.79–13.25], 3.03 [2.12–4.56], *p* = 0.000). 26 Notably, a significant increase in neutrophil count and a parallel decrease in lymphocyte count were observed in the AAD group, while only a decrease in lymphocyte count was seen in the chronic AD group. It seems that after an initial transient neutrophil response, a signiﬁcant and persistent lymphopenia is observed, particularly in patients with the worst prognosis. The optimal cutoff point for the NLR to distinguish AAD was 5.67 (AUC [95% CI]: 0.877 [0.844–0.905]). 26 NLR was significantly increased in patients with Type A compared with those with Type B AAD ( *p* < 0.001). 26

Recently, the systemic inflammatory response index, which was calculated as the monocyte count × NLR ratio, encompasses functions of the three WBC cell subtypes, including neutrophils, lymphocytes, and monocytes and has been associated with the short-term and long-term prognosis of AAD patients who underwent emergency open surgery. 27

### Platelets

#### Platelet Count and Mean Platelet Volume

Marked thrombocytopenia has been universally observed in all AAD studies. A reduction in PLT count has been proposed to correlate with the excessive consumption of PLT in response to inflammation and thrombosis of the nonendothelialized false lumen during AAD. 28 However, Li et al observed that the time interval between dissection onset and urgent surgery (≥3 days) was associated with higher PLT count and enhanced PLT activation, mainly in response to more severe ischemia–reperfusion injury and systemic inflammation; specifically, they found higher admission PLTs with increasing attack time up to the 3 days (turning point) (β: 16.2, 95% CI: 12.1, 20.2; *p* < 0.001). 28

Morello et al found that median PLT count was 178 × 10 ^3^ /µL (IQR: 146–217) in patients with AAS and 207 × 10 ^3^ /µL (IQR: 172–255) in CP patients. 9 In ROC analysis, the AUC was 0.64 (95% CI: 0.58–0.69, *p* < 0.001) for PLT count at a cutoff value < 200 × 10 ^3^ /µL, with a sensitivity of 68.2% (58.6–76.7%) and specificity of 56.2% (52.7–59.7%) for the diagnosis of AAS among patients at low pretest probability. In the same study group, the estimated risk of AAS was lower in patients fulfilling zero criteria (normal WBC and PLT count) and higher in patients fulfilling two criteria (both WBC count > 9 × 10 ^3^ /µL and PLT count < 200 × 10 ^3^ /µL; *p* < 0.001). 9 Of interest, patients with Stanford Type A AAD presented lower PLT count (170 × 10 ^3^ /µL, IQR: 145–207 vs. 187 × 10 ^3^ /µL, IQR: 158–240; *p* = 0.018) compared with patients with other forms of AAS, whereas WBC did not differ ( *p* = 0.119). 9 Consistent with these results, Zhang et al showed that a greater extent of dissection was associated with a lower PLTs and higher serum CRP. 29

Several markers of PLT size and variability in size as well as in function have been studied in patients with abdominal and thoracic aortic aneurysms compared with age-matched controls. Mean platelet volume (MPV) is an indirect measure of PLT reactivity with the main advantage being the rapid and stable increase; 30 larger platelets are metabolically more active, and have greater prothombotic potential. 30 Sbarouni et al found signiﬁcantly lower PLT counts in AAD compared with chronic aneurysms of the ascending aorta and controls. 15 31 We have also found increased MPV/PLT ratio, mainly resulting from enhanced PLT consumption, in the setting of ﬁbrinolytic overactivity. This is further supported by the signiﬁcant relationship between PLT count and D-dimers in the dissection group. 31 MPV as well as the MPV/PLT ratio were signiﬁcantly lower in patients with more extensive dissection, whereas PLT count was lower and the MPV/PLT ratio was higher in Type A dissection compared with Type B. 31

#### Platelet to Lymphocyte Ratio

Platelet to lymphocyte ratio (PLR) integrates the risk prediction of PLTs and lymphocytes, reflecting the activation of coagulation, inflammatory, and immunomodulatory pathways. 32 Increased PLR, correlated with elevated number and enhanced PLT function along with decreased lymphocytes, has been reported as a risk factor for arterial obstructive diseases and vascular injury. 33 We found higher PLR in AAD compared with both aortic aneurysms and controls ( *p* < 0.001), with no difference between the last two. 34 The best cutoff value of PLR to predict dissection was 159 with 53% sensitivity and 86% specificity, whereas Bedel et al showed that the best PLR cutoff value was 195.8, with 76.5% sensitivity and 78.1% specificity. 35 Although the reported levels of sensitivity differed, high specificity seems to be consistent.

The thromboinflammatory index (STI = PLT/WBC) and the systemic immune-inflammation index (SII = PLT × NLR) have been investigated as more comprehensive hematological markers, combining changes in multiple cell types and better representing the current level along the inflammation and immune axis. However, the potential predictive value of the above PLT-derived indices needs further research. 36 37

### Red Blood Cell Count

#### Hemoglobin

Anemia leads to chronic tissue and organ hypoxia, contributing not only to hemodynamic disturbances, but also to chronic inflammatory response and vascular damage thus increasing the risk of developing AAD. 38 39 However, there are not enough data to suggest an association between levels of hemoglobin (Hb) and AAD. Previous studies had observed an inverse relationship between chronic anemia and both, aortic diameter and mortality in patients with abdominal aortic aneurysms via several effects of the anemia-induced erythropoietin. 40 Iron deficiency was associated with the development of aortic medial degeneration in 200 patients with aortic dissection or aneurysm compared with 60 hypertensive patients and with no significant decrease in Hb between the groups. 41 In the AAD group, the concentrations of hematocrit were signiﬁcantly lower than the acute myocardial infarction group as shown by Hazui et al; but it was impossible to discriminate between the groups using cutoff values (AUC = 0.336 [95% CI: 0.204–0.468]). 7 Likewise, our study demonstrated markedly lower Hb levels in patients with Type A AAD compared with both chronic aortic aneurysms and normal subjects (12.6 [IQR: 11.1–13.7] vs. 14.0 [IQR: 13.1–15.2] vs.13.9 [IQR: 12.8–15.0]; *p* < 0.001). 15

#### Red Cell Distribution Width

Red cell distribution width (RDW), a measure of the variability of the size of circulating erythrocytes, may be important in the diagnosis and prognosis of various cardiovascular disorders. Only a limited number of studies have investigated the associations among impaired erythrocyte maturation, RDW, and aneurysm; therefore, the clinical usefulness of RDW alone is unclear. In a population-based study, the cumulative incidence rate of abdominal aortic aneurysms was 61% for patients in the highest quartile of RDW as compared with those with the lowest quartile (hazard ratio = 1.61, CI = 1.20, 2.12) after adjustments for WBC, and this difference between quartiles increased with time ( *p* < 0.001). 42 Our group also evaluated RDW and RDW/PLTs (RPR) in AAD compared with uncomplicated aneurysms of the ascending aorta and controls. 34 We found no significant difference in RDW when corrected for Hb values ( *p* = 0.131), but the RPR index was significantly higher in AAD even after adjustment for Hb ( *p* = 0.001). 34

## Limitations of Use

Most studies conducted until now are nonrandomized, single- or two-center observational investigations with retrospective design. Small sample sizes have limited the statistical power of the analyses and not allowed the subgroup analysis according to type of dissection (Types A and B) or the control disease (acute myocardial infarction, angina, and pulmonary embolism). These clinical entities come under consideration in critically ill patients. Each entity requires rapid and accurate diagnostic workup as management differs for each of these diagnoses. Furthermore, the majority of the studied control groups matched healthy individuals and no other CP patients; this shortcoming may had reduced the effectiveness of the results. Because of the retrospective design, the associations observed do not simply equate a cause–effect relationship. Not it is clear that the findings are a specific consequence of the dissection process itself. It should also be noted that majority of studies have excluded participants who were diagnosed with variant AAS pathologies such as intramural hematoma or penetrating atherosclerotic ulcer. Nor were subjects with possible primary hematological disorders identified and excluded from analysis.

More basic experimental research remains to be conducted in order to elucidate the molecular mechanisms that define the associations among AAD, symptoms and the hematologic biomarkers discussed in this paper. Larger sample size and multicenter trials are further needed to clarify and generalize the results.

## Concluding Remarks and Future Considerations

**Fig. 1:**
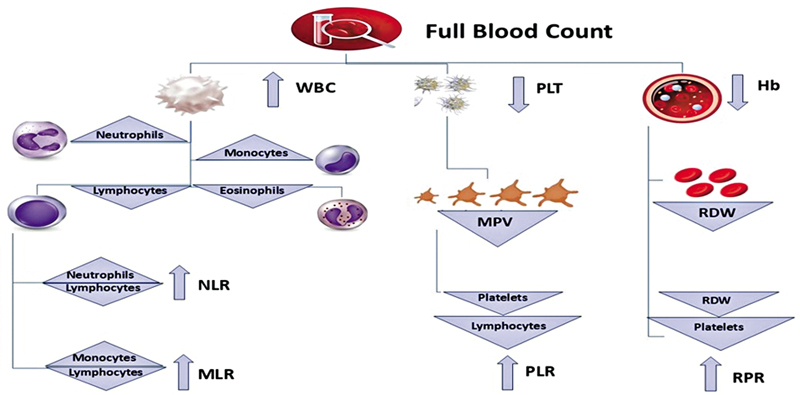
A diagram illustrating the main changes of full blood count -derived indices associated with acute aortic dissection.

AAD is a life-threatening condition and its prompt diagnosis determines the outcome. There is great need for rapid diagnostic tests, applicable in the prehospital setting or the emergency room. Based upon the clinical evidence, three FBC parameters of WBC, PLT counts, and NLR appear to be more relevant and efficient than other parameters ( Table 2 , Fig. 1 ). Leukocytosis mainly due to an increase in neutrophils and marked thrombocytopenia are the most common observations in patients with AAD. NLR is a powerful biomarker to predict mortality in AAD. More studies are needed to examine the usefulness of multiple FBC elements, separately or combined, to predict AAD. An expanded FBC-derived risk score also comprising Hb, MPV, and RDW could provide substantially greater predictive value ( Table 2 ). FBC with differentials is always obtained on admission to the ED. In combination with D-dimer, this biomarker panel could contribute substantially to the diagnostic algorithm and point toward the most efficient imaging modality and optimal management.

**Table 2:** Summary of the main findings for each full blood count component

White blood cell count (WBC)	✓ Increased levels in AAD even in low probability cases compared with other chest pain groups; this seems to be more valid for up to 48 hours as there is an inverse relationship with the time period from disease onset to hospital admission ✓ Significant association with aortic diameter and the extent of dissection
Neutrophils	✓ Increased levels in AAD even in low probability cases compared with other chest pain groups at the time interval of 2–24 h after symptom onset ✓ Higher neutrophil percentage in patients with Type A AAD compared with both chronic aortic aneurysms and normal subjects
Lymphocytes	✓ Decreased levels in AAD compared with those of patients with chronic heart disease and normal volunteers
Monocytes	✓ Elevated levels in AAD compared with those of patients with chronic heart disease and normal volunteers
Eosinophils	✓ Decreased levels in AAD compared with healthy controls
Lymphocyte to monocyte ratio (LMR) or monocyte to lymphocyte ratio (MLR)	✓ Decreased LMR or increased MLR have been associated with high incidence of in-hospital mortality in AAD patients
Neutrophil to lymphocyte ratio (NLR)	✓ NLR was predictive of the diagnosis of AAD with high sensitivity and even higher specificity at cutoff values above 4.6 at presentation ✓ NLR was significantly increased in patients with Type A compared with those with Type B AAD ✓ Elevated NLR in AAD due to combined neutrophilia and lymphopenia; after an initial transient neutrophil response, a signiﬁcant and persistent lymphopenia is observed
Platelets (PLTs)	✓ Decreased PLT count up to the 3 d from dissection onset ✓ High sensitivity and specificity of PLT at a cutoff value <200 × 10 ^3^ /µL for the diagnosis of AAS amongst patients at low pretest probability. ✓ Lower PLT count was presented in patients with Stanford Type A AAD compared with Type B and was associated with a greater extent of dissection ✓ MPV/PLT ratio were signiﬁcantly lower in patients with more extensive dissection whereas PLT count was lower and the MPV/PLT ratio was higher in Stanford Type A AAD compared with Type B ✓ RDW/PLTs was significantly higher in AAD compared with uncomplicated aneurysms of the ascending aorta and controls even after adjustment for Hb
Platelet to lymphocyte ratio (PLR)	✓ Significantly higher PLR in AAD compared with both aortic aneurysms and controls, with no difference between the last two
Hemoglobin (Hb)	✓ Hb levels seem to be lower in patients with Type A AAD compared with both chronic aortic aneurysms and normal subjects

Abbreviations: AAD, acute aortic dissection; FBC, full blood count; MPV, mean platelet volume; RDW, red cell distribution width.
